# Supplementary material for: Artificial Intelligence in mental health and the biases of language based models
Source: PLoS One. 2020 Dec 17;15(12):e0240376. doi: 10.1371/journal.pone.0240376 (PMC7745984; doi:10.1371/journal.pone.0240376)
Supplement: S1 Appendix — (DOCX) [file pone.0240376.s001.docx]

**Appendix 1: MeSH terms and Results of Literature Review**

**Table 1.0** Databases, MeSH terms and Keywords used for literature search

*Carried out from 1^st^ November 2019 – 1^st^ January 2020.

| **Data Base** | **KeyWords** | **Results** |
| --- | --- | --- |
| **(1) PubMed** | (("Natural Language Processing"[Mesh] OR "natural language processing")) AND ((("Mental Health"[Mesh]) OR (("Mental Disorders"[Mesh])) OR ((psychiatr*)) OR ((psychol*))) AND (("ethics" OR "Ethics"[Mesh] OR (ethic*)))) | = **17 articles** |
| **(2) ArXiv** | (([terms: AND all=Natural Language Processing; AND all=psych*;](https://arxiv.org/search/advanced?terms-0-operator=AND&terms-0-term=Natural+Language+Processing&terms-0-field=all&terms-1-operator=AND&terms-1-term=psych%2A&terms-1-field=all&terms-2-operator=AND&terms-2-term=bias&terms-2-field=all&classification-physics_archives=all&classification-include_cross_list=include&date-filter_by=all_dates&date-year=&date-from_date=&date-to_date=&date-date_type=submitted_date&abstracts=show&size=50&order=-announced_date_first) )) & (([terms: AND all=Natural Language Processing; AND all=mental health](https://arxiv.org/search/advanced?terms-0-operator=AND&terms-0-term=Natural+Language+Processing&terms-0-field=all&terms-1-operator=AND&terms-1-term=mental+health&terms-1-field=all&classification-physics_archives=all&classification-include_cross_list=include&date-filter_by=all_dates&date-year=&date-from_date=&date-to_date=&date-date_type=submitted_date&abstracts=show&size=50&order=-announced_date_first))) | **= 11 articles** |
| **(3) Scopus** | ((("Natural Language Processing" OR "natural language processing") AND ("Mental Health" OR "Mental Disorders" OR psychiatr* OR "mental illness" OR psycholog*) AND ("Ethics” OR ethic*))) | **= 2 articles** |
| **(4) ACL Anthology** | order: -announced_date_first; size: 50; terms: AND abstract=Natural Language Processing; AND abstract=mental health; OR title=psychiatry | **= 8 articles** |
| **(5) Engineering Village** | ((("Natural Language Processing" OR "natural language processing") AND ("Mental Health" OR "Mental Disorders" OR psychiatr* OR "mental illness" OR psycholog*) AND ("Ethics” OR ethic*)) | **= 1 article**  (This result was a crossover and therefore was excluded. ) |
| **(6)** [**Association for Computing Machinery (ACM)**](https://www.acm.org/) | (("Natural Language Processing" OR "natural language processing") AND ("Mental Health" OR "Mental Disorders" OR psych* OR "mental illness") | **= 14 articles** |
| **Total Articles** |  | **52 Articles** |
