# Supplementary material for: Artificial Intelligence in mental health and the biases of language based models
Source: PLoS One. 2020 Dec 17;15(12):e0240376. doi: 10.1371/journal.pone.0240376 (PMC7745984; doi:10.1371/journal.pone.0240376)
Supplement: S2 Appendix — (DOCX) [file pone.0240376.s002.docx]

**Appendix 2: Articles included in Literature Review**

*Carried out from 1^st^ November 2019 – 1^st^ January 2020.

**PubMed** (<https://pubmed.ncbi.nlm.nih.gov/>)

1. [**Empathy Bot: Conversational Service for Psychiatric Counseling with Chat Assistant.**](https://www.ncbi.nlm.nih.gov/pubmed/29295322)

*Oh, Kyo-Joong, et al. “Empathy Bot: Conversational Service for Psychiatric Counseling with Chat Assistant.” Studies in Health Technology and Informatics, vol. 245, 2017, p. 1235.*

1. **Gaining insights from social media language: Methodologies and challenges.**

*Kern, Margaret L., et al. “Gaining Insights from Social Media Language: Methodologies and Challenges.” Psychological Methods, vol. 21, no. 4, 2016, pp. 507–25. PubMed, doi:10.1037/met0000091.*

1. **Identification of suicidal behavior among psychiatrically hospitalized adolescents using natural language processing and machine learning of electronic health records.**

*Carson, Nicholas J., et al. “Identification of Suicidal Behavior among Psychiatrically Hospitalized Adolescents Using Natural Language Processing and Machine Learning of Electronic Health Records.” PloS One, vol. 14, no. 2, 2019, p. e0211116. PubMed, doi:10.1371/journal.pone.0211116.*

1. **Monitoring suicidal patients in primary care using electronic health records.**

*Anderson, Heather D., et al. “Monitoring Suicidal Patients in Primary Care Using Electronic Health Records.” Journal of the American Board of Family Medicine: JABFM, vol. 28, no. 1, Feb. 2015, pp. 65–71. PubMed, doi:10.3122/jabfm.2015.01.140181.*

1. **Text Classification to Inform Suicide Risk Assessment in Electronic Health Records.**

*Bittar, André, et al. “Text Classification to Inform Suicide Risk Assessment in Electronic Health Records.” Studies in Health Technology and Informatics, vol. 264, Aug. 2019, pp. 40–44. PubMed, doi:10.3233/SHTI190179.*

1. **Novel Use of Natural Language Processing (NLP) to Predict Suicidal Ideation and Psychiatric Symptoms in a Text-Based Mental Health Intervention in Madrid.**

*Cook, Benjamin L., et al. “Novel Use of Natural Language Processing (NLP) to Predict Suicidal Ideation and Psychiatric Symptoms in a Text-Based Mental Health Intervention in Madrid.” Computational and Mathematical Methods in Medicine, vol. 2016, 2016, p. 8708434. PubMed, doi:10.1155/2016/8708434.*

1. **Identifying Suicide Ideation and Suicidal Attempts in a Psychiatric Clinical Research Database using Natural Language Processing.**

*Fernandes, Andrea C., et al. “Identifying Suicide Ideation and Suicidal Attempts in a Psychiatric Clinical Research Database Using Natural Language Processing.” Scientific Reports, vol. 8, no. 1, 09 2018, p. 7426. PubMed, doi:10.1038/s41598-018-25773-2.*

1. **Research Domain Criteria scores estimated through natural language processing are associated with risk for suicide and accidental death.**

*McCoy, Thomas H., et al. “Research Domain Criteria Scores Estimated through Natural Language Processing Are Associated with Risk for Suicide and Accidental Death.” Depression and Anxiety, vol. 36, no. 5, 2019, pp. 392–99. PubMed, doi:10.1002/da.22882.*

1. **A Controlled Trial Using Natural Language Processing to Examine the Language of Suicidal Adolescents in the Emergency Department.**

*Pestian, John P., et al. “A Controlled Trial Using Natural Language Processing to Examine the Language of Suicidal Adolescents in the Emergency Department.” Suicide & Life-Threatening Behavior, vol. 46, no. 2, Apr. 2016, pp. 154–59. PubMed, doi:10.1111/sltb.12180.*

1. **Suicide Note Classification Using Natural Language Processing: A Content Analysis**

Pestian, John, et al. “Suicide Note Classification Using Natural Language Processing: A Content Analysis.” Biomedical Informatics Insights, vol. 2010, no. 3, Aug. 2010, pp. 19–28. PubMed, doi:10.4137/bii.s4706.

1. **A hybrid approach to sentiment sentence classification in suicide notes.**

*Sohn, Sunghwan, et al. “A Hybrid Approach to Sentiment Sentence Classification in Suicide Notes.” Biomedical Informatics Insights, vol. 5, no. Suppl. 1, 2012, pp. 43–50. PubMed, doi:10.4137/BII.S8961.*

1. **Identifying Suicidal Adolescents from Mental Health Records Using Natural Language Processing.**

*Velupillai, Sumithra, et al. “Identifying Suicidal Adolescents from Mental Health Records Using Natural Language Processing.” Studies in Health Technology and Informatics, vol. 264, Aug. 2019, pp. 413–17. PubMed, doi:10.3233/SHTI190254.*

1. **Using text mining to extract depressive symptoms and to validate the diagnosis of major depressive disorder from electronic health records.**

*Wu, Chi-Shin, et al. “Using Text Mining to Extract Depressive Symptoms and to Validate the Diagnosis of Major Depressive Disorder from Electronic Health Records.” Journal of Affective Disorders, vol. 260, Jan. 2020, pp. 617–23. PubMed, doi:10.1016/j.jad.2019.09.044.*

1. **Psychiatric stressor recognition from clinical notes to reveal association with suicide.**

*Zhang, Yaoyun, et al. “Psychiatric Stressor Recognition from Clinical Notes to Reveal Association with Suicide.” Health Informatics Journal, vol. 25, no. 4, 2019, pp. 1846–62. PubMed, doi:10.1177/1460458218796598.*

1. **Screening pregnant women for suicidal behavior in electronic medical records: diagnostic codes vs. clinical notes processed by natural language processing.**

*Zhong, Qiu-Yue, et al. “Screening Pregnant Women for Suicidal Behavior in Electronic Medical Records: Diagnostic Codes vs. Clinical Notes Processed by Natural Language Processing.” BMC Medical Informatics and Decision Making, vol. 18, no. 1, 29 2018, p. 30. PubMed, doi:10.1186/s12911-018-0617-7.*

1. **Use of natural language processing in electronic medical records to identify pregnant women with suicidal behavior: towards a solution to the complex classification problem.**

*Zhong, Qiu-Yue, et al. “Use of Natural Language Processing in Electronic Medical Records to Identify Pregnant Women with Suicidal Behavior: Towards a Solution to the Complex Classification Problem.” European Journal of Epidemiology, vol. 34, no. 2, Feb. 2019, pp. 153–62. PubMed, doi:10.1007/s10654-018-0470-0.*

1. **Natural Language Processing of Social Media as Screening for Suicide Risk.**

*Coppersmith, Glen, et al. “Natural Language Processing of Social Media as Screening for Suicide Risk.” Biomedical Informatics Insights, vol. 10, 2018, p. 1178222618792860. PubMed, doi:10.1177/1178222618792860*

**ArXiv** (<https://arxiv.org/>)

1. **Emotion Recognition in Conversation: Research Challenges, Datasets, and Recent Advances.**

*Poria, Soujanya, et al. “Emotion Recognition in Conversation: Research Challenges, Datasets, and Recent Advances.” ArXiv:1905.02947 [Cs], May 2019. arXiv.org, http://arxiv.org/abs/1905.02947.*

1. **Distinguishing Clinical Sentiment: The Importance of Domain Adaptation in Psychiatric Patient Health Records.**

*Holderness, Eben, et al. “Distinguishing Clinical Sentiment: The Importance of Domain Adaptation in Psychiatric Patient Health Records.” ArXiv:1904.03225 [Cs], Apr. 2019. arXiv.org, http://arxiv.org/abs/1904.03225.*

1. **Emotional Intensity analysis in Bipolar subjects.**

*Carrillo, Facundo, et al. “Emotional Intensity Analysis in Bipolar Subjects.” ArXiv:1606.02231 [Cs, Stat], June 2016. arXiv.org, http://arxiv.org/abs/1606.02231*

1. **Large-scale Analysis of Counseling Conversations: An Application of Natural Language Processing to Mental Health**.

*Althoff, Tim, et al. Large-Scale Analysis of Counseling Conversations: An Application of Natural Language Processing to Mental Health. May 2016. arxiv.org, https://arxiv.org/abs/1605.04462v3.*

1. **Measuring Depression Symptom Severity from Spoken Language and 3D Facial Expressions.**

*Haque, Albert, et al. “Measuring Depression Symptom Severity from Spoken Language and 3D Facial Expressions.” ArXiv:1811.08592 [Cs, Eess], Nov. 2018. arXiv.org, http://arxiv.org/abs/1811.08592.*

1. **Deep learning for language understanding of mental health concepts derived from Cognitive Behavioural Therapy.**

*Rojas-Barahona, Lina, et al. “Deep Learning for Language Understanding of Mental Health Concepts Derived from Cognitive Behavioural Therapy.” ArXiv:1809.00640 [Cs], Sept. 2018. arXiv.org, http://arxiv.org/abs/1809.00640*

1. [**SMHD: a Large-Scale Resource for Exploring Online Language Usage for Multiple Mental Health Conditions**](https://www.aclweb.org/anthology/C18-1126.pdf)**.**

*Cohan, Arman, et al. “SMHD: A Large-Scale Resource for Exploring Online Language Usage for Multiple Mental Health Conditions.” ArXiv:1806.05258 [Cs], July 2018. arXiv.org, http://arxiv.org/abs/1806.05258.*

1. [**A Computational Linguistic Study of Personal Recovery in Bipolar Disorder**](https://www.aclweb.org/anthology/P19-2003.pdf)

*Jagfeld, Glorianna. “A Computational Linguistic Study of Personal Recovery in Bipolar Disorder.” ArXiv:1906.01010 [Cs], June 2019. arXiv.org, http://arxiv.org/abs/1906.01010.*

1. [**Semantic Characteristics of Schizophrenic Speech**](https://www.aclweb.org/anthology/W19-3010.pdf)

*Bar, Kfir, et al. “Semantic Characteristics of Schizophrenic Speech.” ArXiv:1904.07953 [Cs], Apr. 2019. arXiv.org, http://arxiv.org/abs/1904.07953.*

1. **Utilizing Neural Networks and Linguistic Metadata for Early Detection of Depression Indications in Text Sequences**

*Trotzek, Marcel, et al. “Utilizing Neural Networks and Linguistic Metadata for Early Detection of Depression Indications in Text Sequences.” IEEE Transactions on Knowledge and Data Engineering, vol. 32, no. 3, Mar. 2020, pp. 588–601. arXiv.org, doi:10.1109/TKDE.2018.2885515.*

1. **SECNLP: A Survey of Embeddings in Clinical Natural Language Processing**

*KS, Kalyan, and S. Sangeetha. “SECNLP: A Survey of Embeddings in Clinical Natural Language Processing.” Journal of Biomedical Informatics, vol. 101, Jan. 2020, p. 103323. arXiv.org, doi:10.1016/j.jbi.2019.103323.*

**Scopus (**[https://www.scopus.com/)](https://www.scopus.com/search/form.uri?display=basic)

1. [**The chatbot feels you - A counseling service using emotional response generation**](https://www-scopus-com.proxy.library.upenn.edu/record/display.uri?eid=2-s2.0-85017666234&origin=resultslist&sort=plf-f&src=s&st1=%28%28%28%22Natural+Language+Processing%22+OR+%22natural+language+processing%22%29+AND+%28%22Mental+Health%22+OR+%22Mental++Disorders%22+OR+psychiatr*+OR+%22mental+illness%22+OR+psycholog*%29+AND+%28%22Ethics%22+OR+ethic*%29%29%29&st2=&sid=f4349cd1d179bc2e912c93bc5cb0b6b8&sot=b&sdt=b&sl=201&s=TITLE-ABS-KEY%28%28%28%28%22Natural+Language+Processing%22+OR+%22natural+language+processing%22%29+AND+%28%22Mental+Health%22+OR+%22Mental++Disorders%22+OR+psychiatr*+OR+%22mental+illness%22+OR+psycholog*%29+AND+%28%22Ethics%22+OR+ethic*%29%29%29%29&relpos=4&citeCnt=7&searchTerm=)

*Lee, D., Oh, K.-J., Choi, H.-J. The chatbot feels you - A counseling service using emotional response generation (2017) IEEE International Conference on Big Data and Smart Computing, BigComp 2017, art. no. 7881752, pp. 437-440. https://www.scopus.com/inward/record.uri?eid=2-s2.0-85017666234&doi=10.1109%2fBIGCOMP.2017.7881752&partnerID=40&md5=27bec8c161dbdbc47bab220de5f2fe1d*

1. **Text-Based Detection and Understanding of Changes in Mental Health**

*Li, Y., Mihalcea, R., Wilson, S.R. Text-based detection and understanding of changes in mental health (2018) Lecture Notes in Computer Science (including subseries Lecture Notes in Artificial Intelligence and Lecture Notes in Bioinformatics), 11186 LNCS, pp. 176-188.*

*https://www.scopus.com/inward/record.uri?eid=2-s2.0-85057226097&doi=10.1007%2f978-3-030-01159-8_17&partnerID=40&md5=7ce96c297fa115236e52c910ba88739f*

**ACL Anthology** (<https://www.aclweb.org/anthology/>)

1. **CLPsych 2019 Shared Task: Predicting the Degree of Suicide Risk in Reddit Posts**

*Zirikly, Ayah, et al. “CLPsych 2019 Shared Task: Predicting the Degree of Suicide Risk in Reddit Posts.” Proceedings of the Sixth Workshop on Computational Linguistics and Clinical Psychology, Association for Computational Linguistics, 2019, pp. 24–33. ACLWeb, doi:10.18653/v1/W19-3003*

1. **Depression and Self-Harm Risk Assessment in Online Forums**

*Yates, Andrew, et al. “Depression and Self-Harm Risk Assessment in Online Forums.” Proceedings of the 2017 Conference on Empirical Methods in Natural Language Processing, Association for Computational Linguistics, 2017, pp. 2968–2978. ACLWeb, doi:10.18653/v1/D17-1322.*

1. [**Towards Developing an Annotation Scheme for Depressive Disorder Symptoms: A Preliminary Study using Twitter Data**](https://www.aclweb.org/anthology/W15-1211.pdf)

*Mowery, Danielle, et al. “Towards Developing an Annotation Scheme for Depressive Disorder Symptoms: A Preliminary Study Using Twitter Data.” Proceedings of the 2nd Workshop on Computational Linguistics and Clinical Psychology: From Linguistic Signal to Clinical Reality, Association for Computational Linguistics, 2015, pp. 89–98. ACLWeb, doi:10.3115/v1/W15-1211.*

1. [**Suicide Risk Assessment with Multi-level Dual-Context Language and BERT**](https://www.aclweb.org/anthology/W19-3005.pdf)

*Matero, Matthew, et al. “Suicide Risk Assessment with Multi-Level Dual-Context Language and BERT.” Proceedings of the Sixth Workshop on Computational Linguistics and Clinical Psychology, Association for Computational Linguistics, 2019, pp. 39–44. ACLWeb, doi:10.18653/v1/W19-3005.*

1. **Exploratory Analysis of Social Media Prior to a Suicide Attempt**

*Coppersmith, Glen, et al. “Exploratory Analysis of Social Media Prior to a Suicide Attempt.” Proceedings of the Third Workshop on Computational Linguistics and Clinical Psychology, Association for Computational Linguistics, 2016, pp. 106–117. ACLWeb, doi:10.18653/v1/W16-0311*

1. [**Expert, Crowdsourced and Machine Assessment of Suicide Risk via Online Postings**](https://www.aclweb.org/anthology/W18-0603.pdf)

*Shing, Han-Chin, et al. “Expert, Crowdsourced, and Machine Assessment of Suicide Risk via Online Postings.” Proceedings of the Fifth Workshop on Computational Linguistics and Clinical Psychology: From Keyboard to Clinic, Association for Computational Linguistics, 2018, pp. 25–36. ACLWeb, doi:10.18653/v1/W18-0603*

1. **CLP Psych: “Shared Task: Depression and PTSD on Twitter”**

*Coppersmith, Glen, et al. “CLPsych 2015 Shared Task: Depression and PTSD on Twitter.” Proceedings of the 2nd Workshop on Computational Linguistics and Clinical Psychology: From Linguistic Signal to Clinical Reality, Association for Computational Linguistics, 2015, pp. 31–39. ACLWeb, doi:10.3115/v1/W15-1204.*

1. **Can adult mental health be predicted by childhood future-self narratives? Insights from the CLPsych 2018 Shared Task.**

*Radford, Kylie, et al. “Can Adult Mental Health Be Predicted by Childhood Future-Self Narratives? Insights from the CLPsych 2018 Shared Task.” Proceedings of the Fifth Workshop on Computational Linguistics and Clinical Psychology: From Keyboard to Clinic, Association for Computational Linguistics, 2018, pp. 126–135. ACLWeb, doi:10.18653/v1/W18-0614.*

**Association for Computing Machinery (ACM)** (<https://dl.acm.org/>)

1. **Semi-Supervised Approach to Monitoring Clinical Depressive Symptoms in Social Media**

*Yazdavar, Amir Hossein, et al. “Semi-Supervised Approach to Monitoring Clinical Depressive Symptoms in Social Media.” Proceedings of the 2017 IEEE/ACM International Conference on Advances in Social Networks Analysis and Mining 2017, Association for Computing Machinery, 2017, pp. 1191–1198. ACM Digital Library, doi:10.1145/3110025.3123028.*

1. **Using natural language processing to classify suicide notes**

*Pestian, John P., et al. “Using Natural Language Processing to Classify Suicide Notes.” Proceedings of the Workshop on Current Trends in Biomedical Natural Language Processing, Association for Computational Linguistics, 2008, pp. 96–97.*

1. **Clustering semantic spaces of suicide notes and newsgroups articles**

*Matykiewicz, P., et al. “Clustering Semantic Spaces of Suicide Notes and Newsgroups Articles.” Proceedings of the Workshop on Current Trends in Biomedical Natural Language Processing, Association for Computational Linguistics, 2009, pp. 179–184.*

1. **Machine Classification and Analysis of Suicide-Related Communication on Twitter**

*Burnap, Pete, et al. “Machine Classification and Analysis of Suicide-Related Communication on Twitter.” Proceedings of the 26th ACM Conference on Hypertext & Social Media, Association for Computing Machinery, 2015, pp. 75–84. ACM Digital Library, doi:10.1145/2700171.2791023.*

1. **A hybrid statistical and semantic model for identification of mental health and behavioral disorders using social network analysis**

*Krishnamurthy, Madan, et al. “A Hybrid Statistical and Semantic Model for Identification of Mental Health and Behavioral Disorders Using Social Network Analysis.” Proceedings of the 2016 IEEE/ACM International Conference on Advances in Social Networks Analysis and Mining, IEEE Press, 2016, pp. 1019–1026.*

1. **"Let Me Tell You About Your Mental Health!": Contextualized Classification of Reddit Posts to DSM-5 for Web-based Intervention**

*Gaur, Manas, et al. “‘Let Me Tell You About Your Mental Health!’: Contextualized Classification of Reddit Posts to DSM-5 for Web-Based Intervention.” Proceedings of the 27th ACM International Conference on Information and Knowledge Management, Association for Computing Machinery, 2018, pp. 753–762. ACM Digital Library, doi:10.1145/3269206.3271732.*

1. **Toward Objective, Multifaceted Characterization of Psychotic Disorders: Lexical, Structural, and Disfluency Markers of Spoken Language.**

*Vail, Alexandria K., et al. “Toward Objective, Multifaceted Characterization of Psychotic Disorders: Lexical, Structural, and Disfluency Markers of Spoken Language.” Proceedings of the 20th ACM International Conference on Multimodal Interaction, Association for Computing Machinery, 2018, pp. 170–178. ACM Digital Library, doi:10.1145/3242969.3243020.*

1. **Mood patterns and affective lexicon access in weblogs.**

*Nguyen, Thin. “Mood Patterns and Affective Lexicon Access in Weblogs.” Proceedings of the ACL 2010 Student Research Workshop, Association for Computational Linguistics, 2010, pp. 43–48.*

1. **Social media as a measurement tool of depression in populations.**

*De Choudhury, Munmun, et al. “Social Media as a Measurement Tool of Depression in Populations.” Proceedings of the 5th Annual ACM Web Science Conference, Association for Computing Machinery, 2013, pp. 47–56. ACM Digital Library, doi:10.1145/2464464.2464480.*

1. **Predicting postpartum changes in emotion and behavior via social media.**

*De Choudhury, Munmun, et al. “Predicting Postpartum Changes in Emotion and Behavior via Social Media.” Proceedings of the SIGCHI Conference on Human Factors in Computing Systems, Association for Computing Machinery, 2013, pp. 3267–3276. ACM Digital Library, doi:10.1145/2470654.2466447.*

1. **Characterizing and predicting postpartum depression from shared facebook data.**

*De Choudhury, Munmun, et al. “Characterizing and Predicting Postpartum Depression from Shared Facebook Data.” Proceedings of the 17th ACM Conference on Computer Supported Cooperative Work & Social Computing, Association for Computing Machinery, 2014, pp. 626–638. ACM Digital Library, doi:10.1145/2531602.2531675.*

1. **Mental Health Discourse on reddit: Self-Disclosure, Social Support, and Anonymity.**

*Choudhury, Munmun De, and Sushovan De. “Mental Health Discourse on Reddit: Self-Disclosure, Social Support, and Anonymity.” Proceedings of the 8th International Conference on Weblogs and Social Media, ICWSM 2014.*

1. **Detecting and Characterizing Mental Health Related Self-Disclosure in Social Media.**

*Balani, Sairam, and Munmun De Choudhury. “Detecting and Characterizing Mental Health Related Self-Disclosure in Social Media.” Proceedings of the 33rd Annual ACM Conference Extended Abstracts on Human Factors in Computing Systems, Association for Computing Machinery, 2015, pp. 1373–1378. ACM Digital Library, doi:10.1145/2702613.2732733.*

1. **Knowledge-aware Assessment of Severity of Suicide Risk for Early Intervention.**

*Gaur, Manas, et al. “Knowledge-Aware Assessment of Severity of Suicide Risk for Early Intervention.” The World Wide Web Conference, Association for Computing Machinery, 2019, pp. 514–525. ACM Digital Library, doi:10.1145/3308558.3313698.*
