# Supplementary material for: Artificial Intelligence in mental health and the biases of language based models
Source: PLoS One. 2020 Dec 17;15(12):e0240376. doi: 10.1371/journal.pone.0240376 (PMC7745984; doi:10.1371/journal.pone.0240376)
Supplement: S3 Appendix — Here we have repeated the analysis performed above with the 50d model to demonstrate consistency of trends. (DOCX) [file pone.0240376.s003.docx]

### Appendix 3

The 50 dimension model is the original model used for creating Graphs 1 - 4 in the manuscript. Here we have repeated the analysis performed above with the 50d model to demonstrate consistency of trends.

**Graph 1**


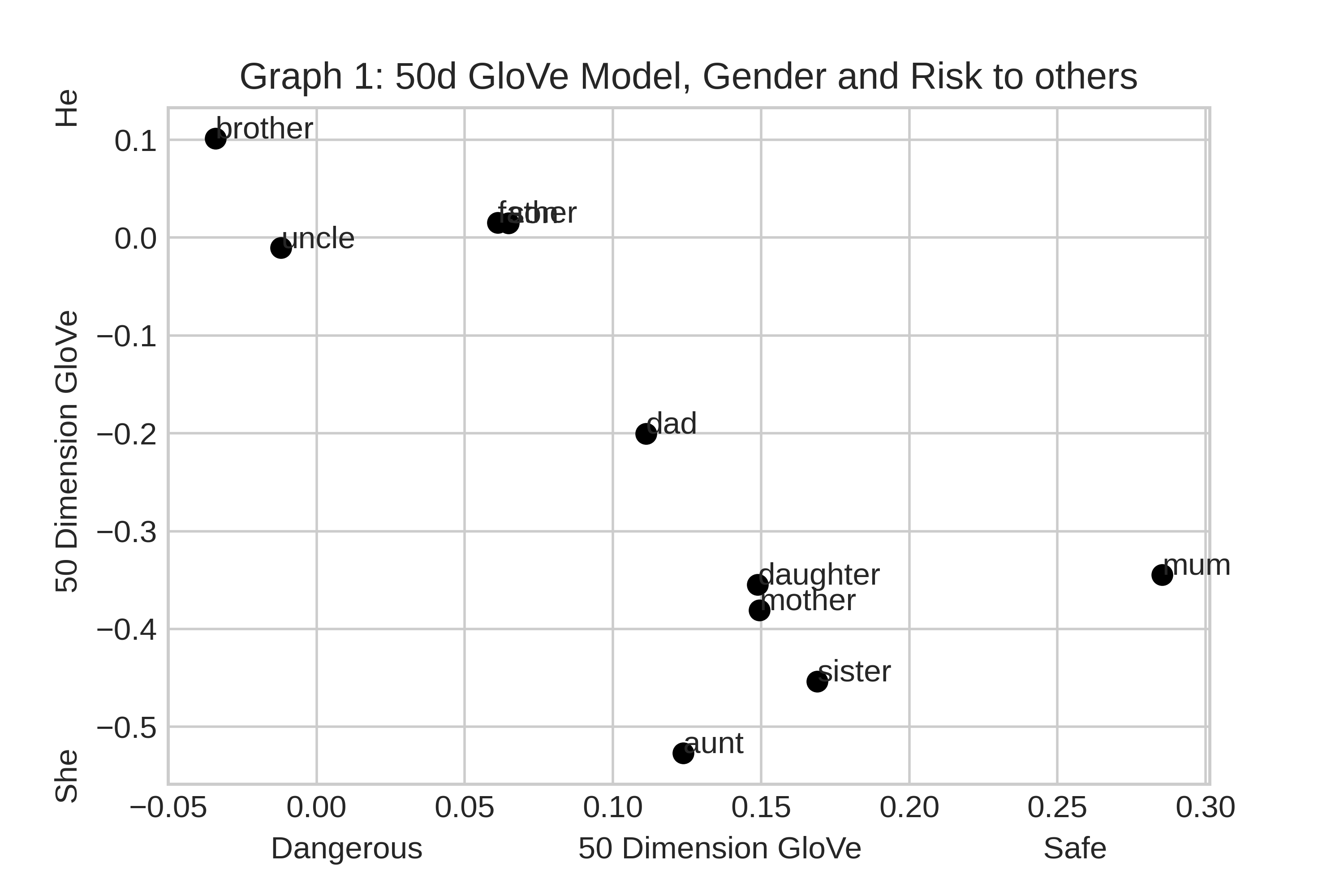


**Graph 1.1 (200d) - Gender Bias and Risk to Others (terms ‘safe’ and ‘dangerous’),**

Graph 1 of the manuscript used the terms ‘safe’ and ‘dangerous’ at either pole of the Y-axis, to assess how different demographic terms (e.g. ‘mum’) relate to the concept of safe. We have repeated this analysis on the 200dimension and 300dimension versions of glove in the graphs below.

**
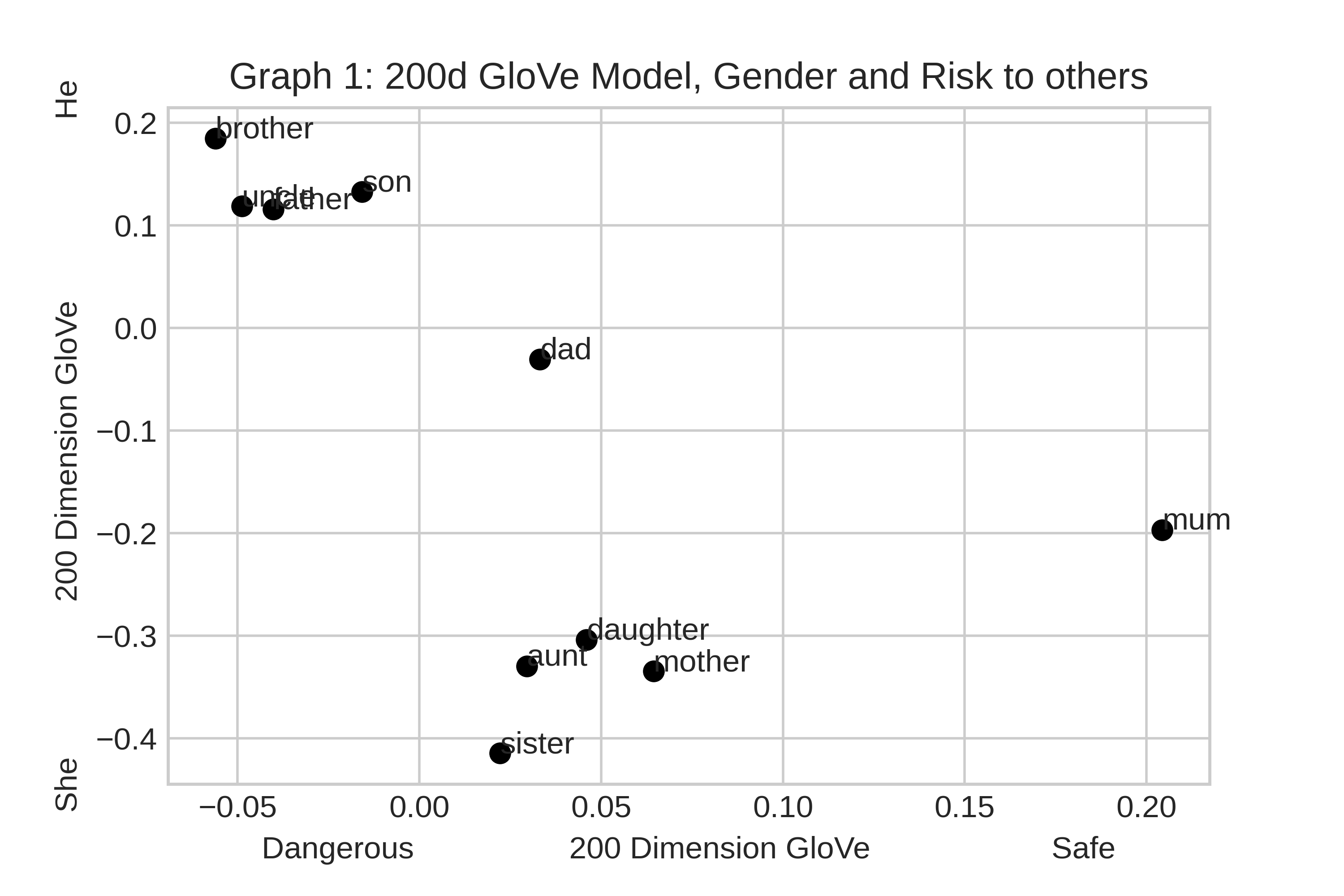
**

**Graph 1.2 (300d) – 300 Dimension Gender Bias and Risk to Others (terms ‘safe’ and ‘dangerous’)**

**
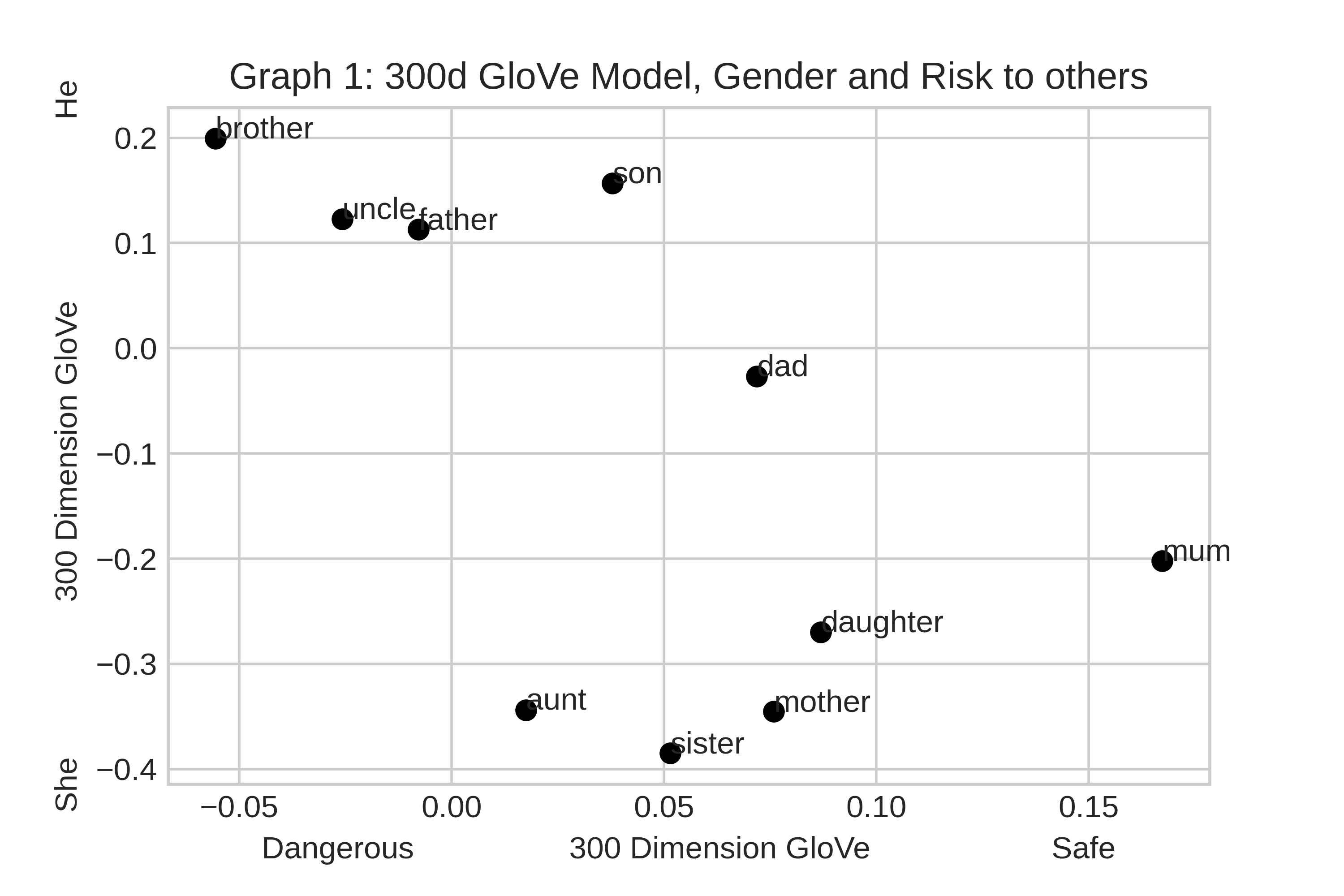
**

**Graph 2**

**
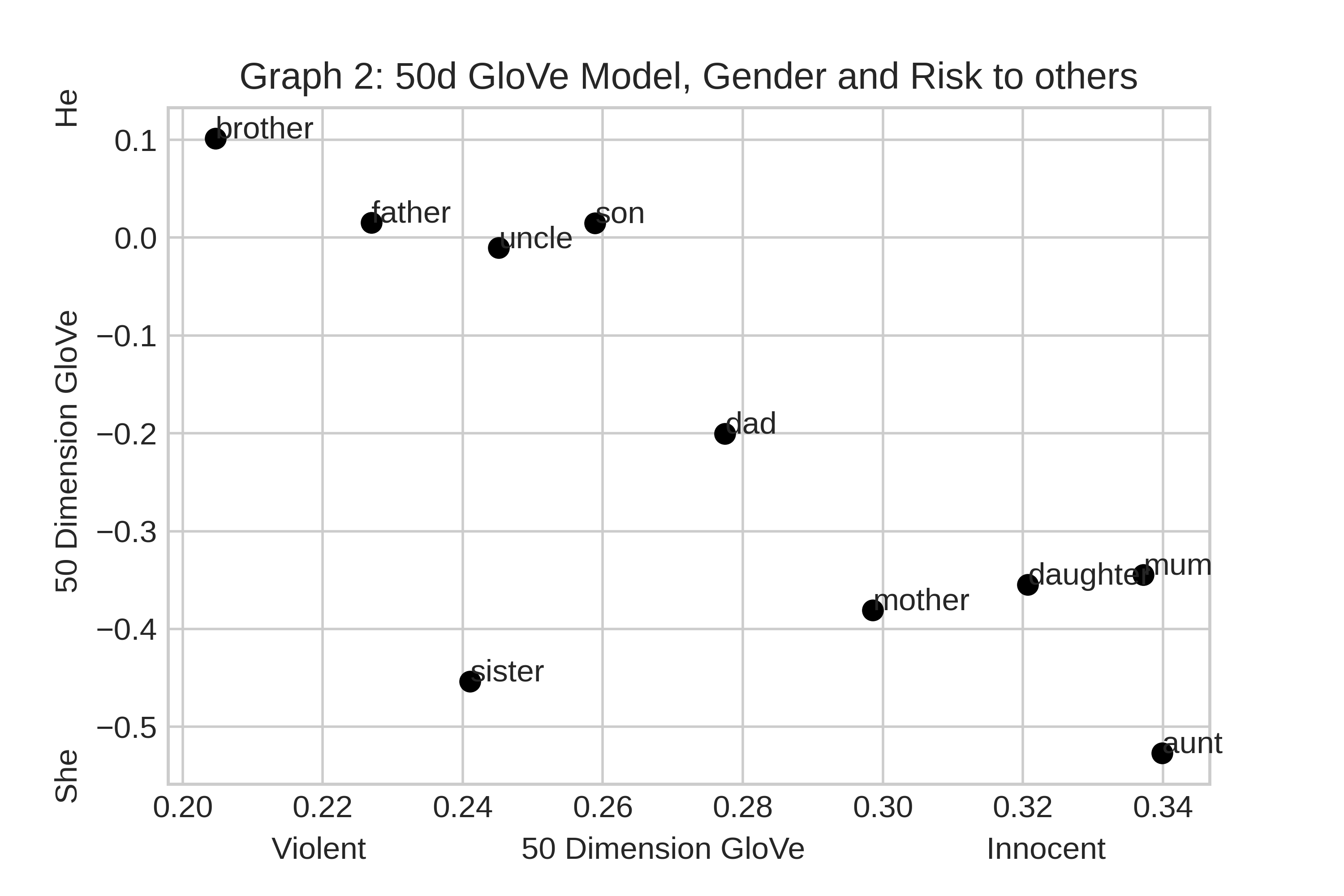
**

**Graph 2.1 (200d) – 200 Dimension Gender Bias and Risk to Others (terms ‘violent’ and ‘innocent’)**

The graph below is an adaptation of the graph above, where we use the alternative terms 'Violent' and 'Innocent' to illustrate the concept of risk. We have repeated this analysis on the 200dimension and 300dimension versions of glove in the graphs below.


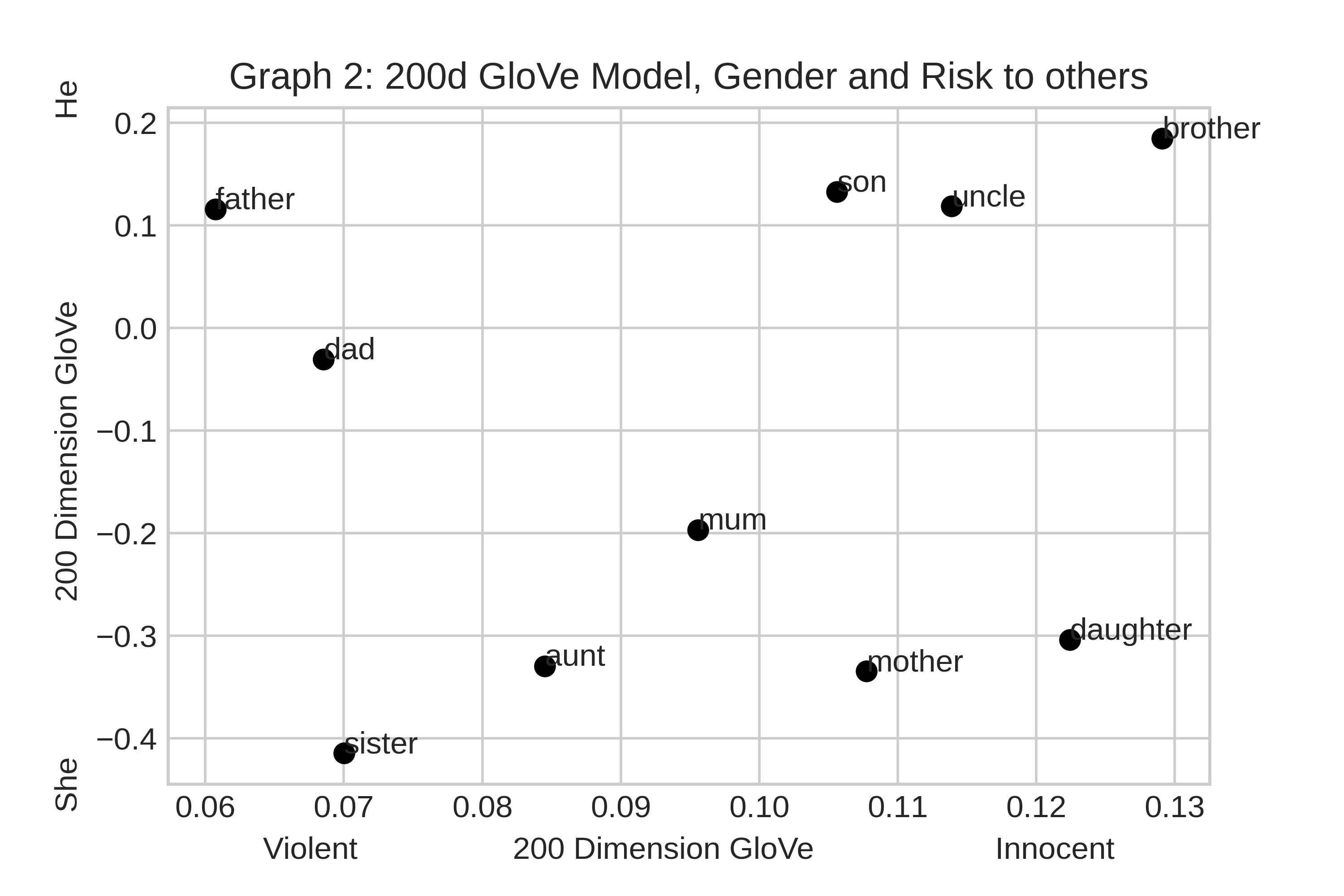


**Graph 2.2 (300d) – 300 Dimension Gender Bias and Risk to Others (terms ‘violent’ and ‘innocent’)**


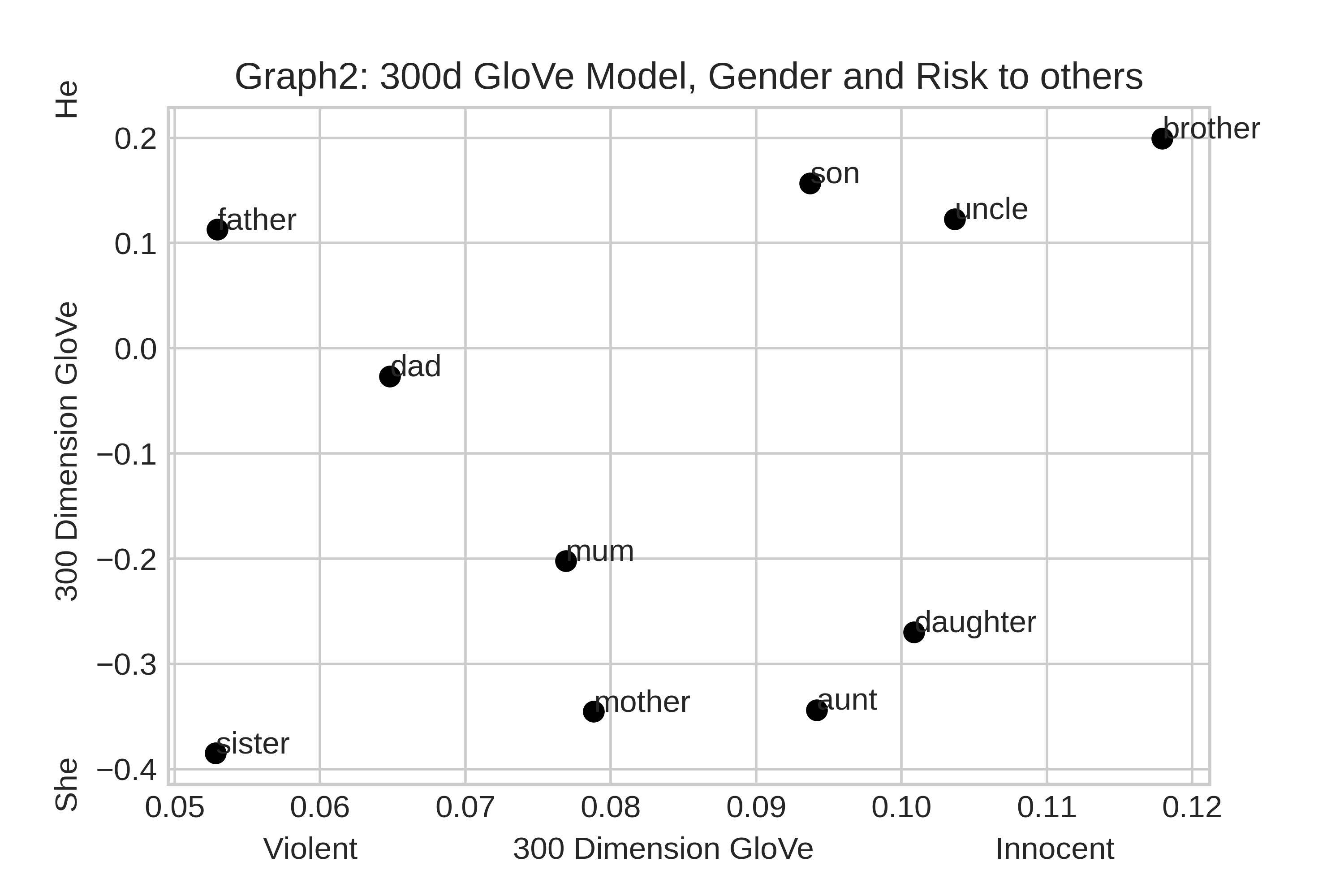


**Graph 3**

**
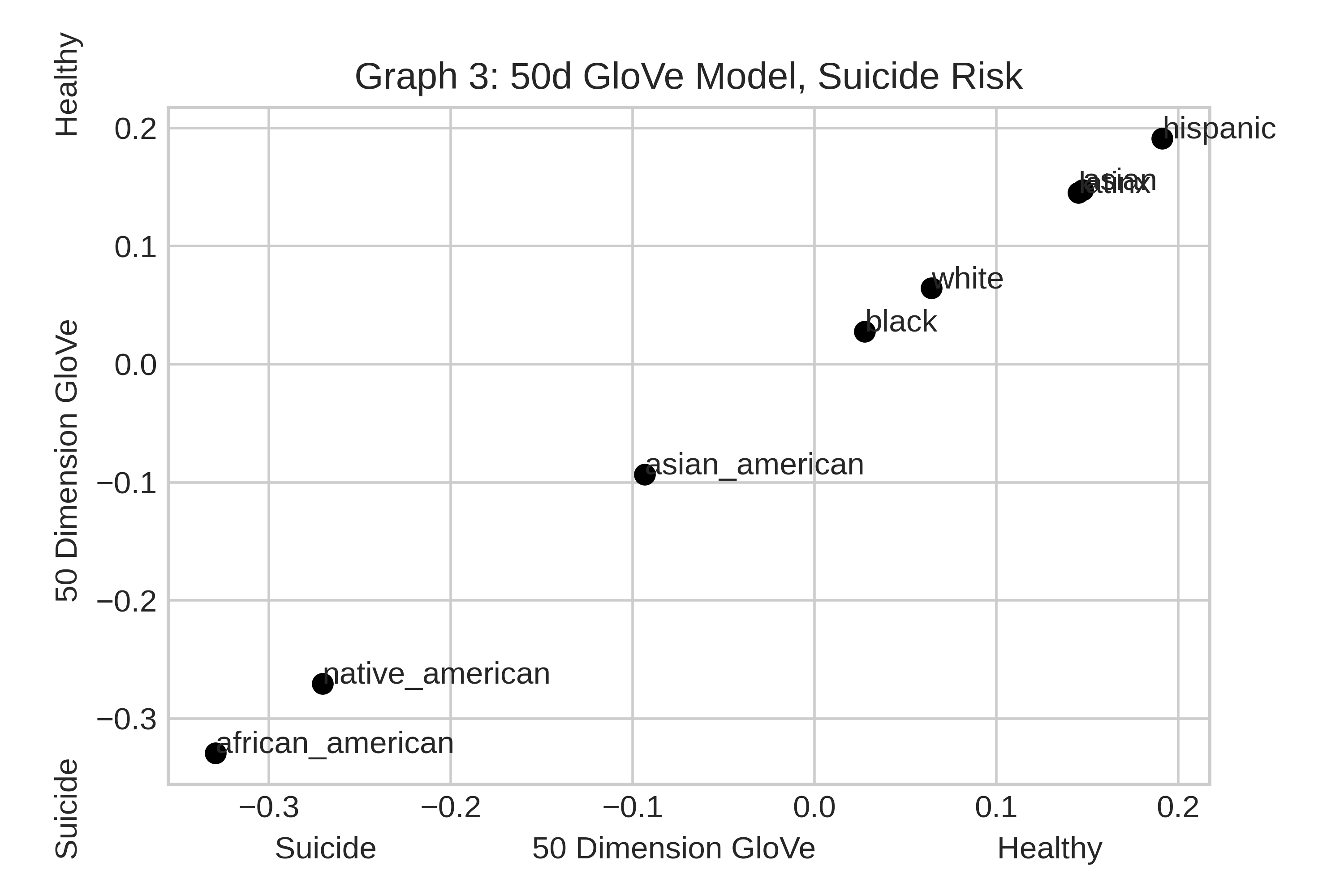
**

**Graph 3.1 (200d): 200 Dimension Racial Bias and Risk To Self ('Suicide' and 'Healthy')**

The graph below is analysis on the 200d analysis of racial labels and the concept of suicide. We can see here that the order of racial label changes from the 50d model, such that the order of terms from 'Suicide' to 'Healthy' is:

(1)black, (2)white, (3) african_american, (4)native_american, (5) latinx, (6)a sian_american, (7) asian, (8) hispanic.

**
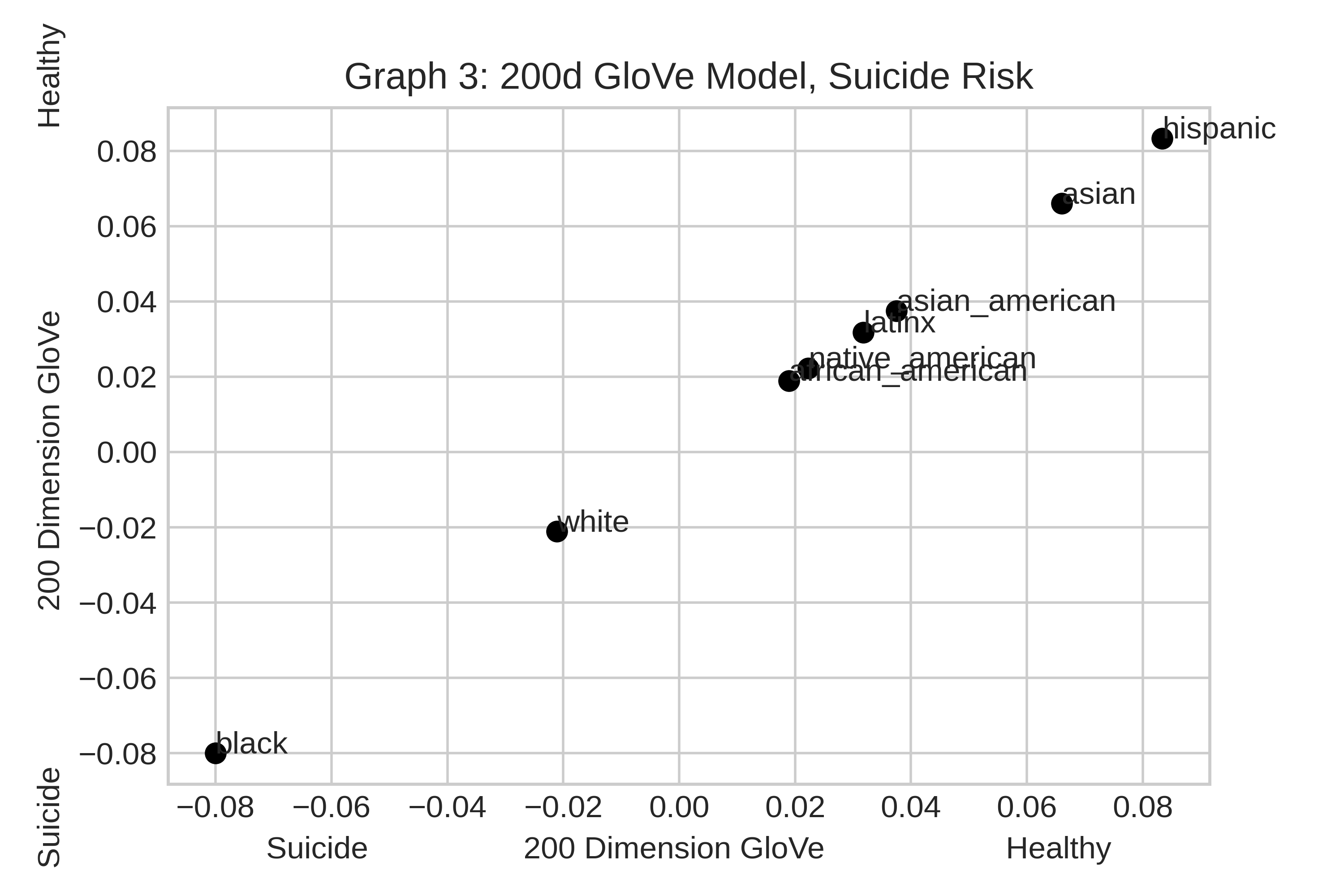
**

**Graph 3.2 (300d): 300 Dimension Racial Bias and Risk To Self ('Suicide' and 'Healthy')**

The graph below is the analysis of the 300 dimension model of GloVe. We can see here that the order of racial label changes, such that the order of terms from 'Suicide' to 'Healthy' is:

(1)black, (2)asian_american, (3) native_american, (4)white, (5)african_american, (6)latinx, (7)asian, (8)hispanic.

The inconsistency of the order of terms illustrates that biases within a model are specific to the type of model (and dimensions) used. This highlights the importance of analysing all models for biases, before using them for a medical or social function.

**
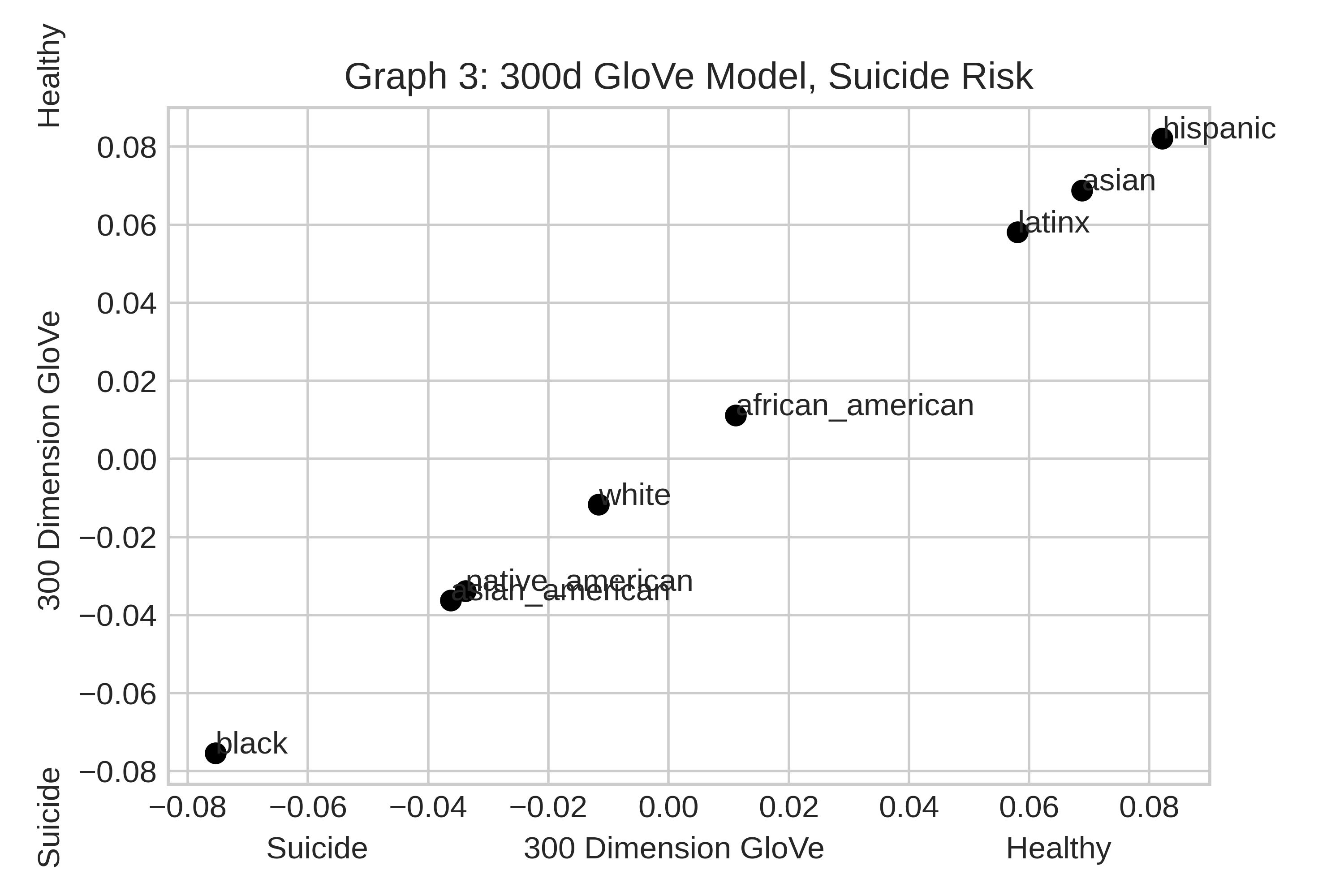
**

**Graph 4**

**
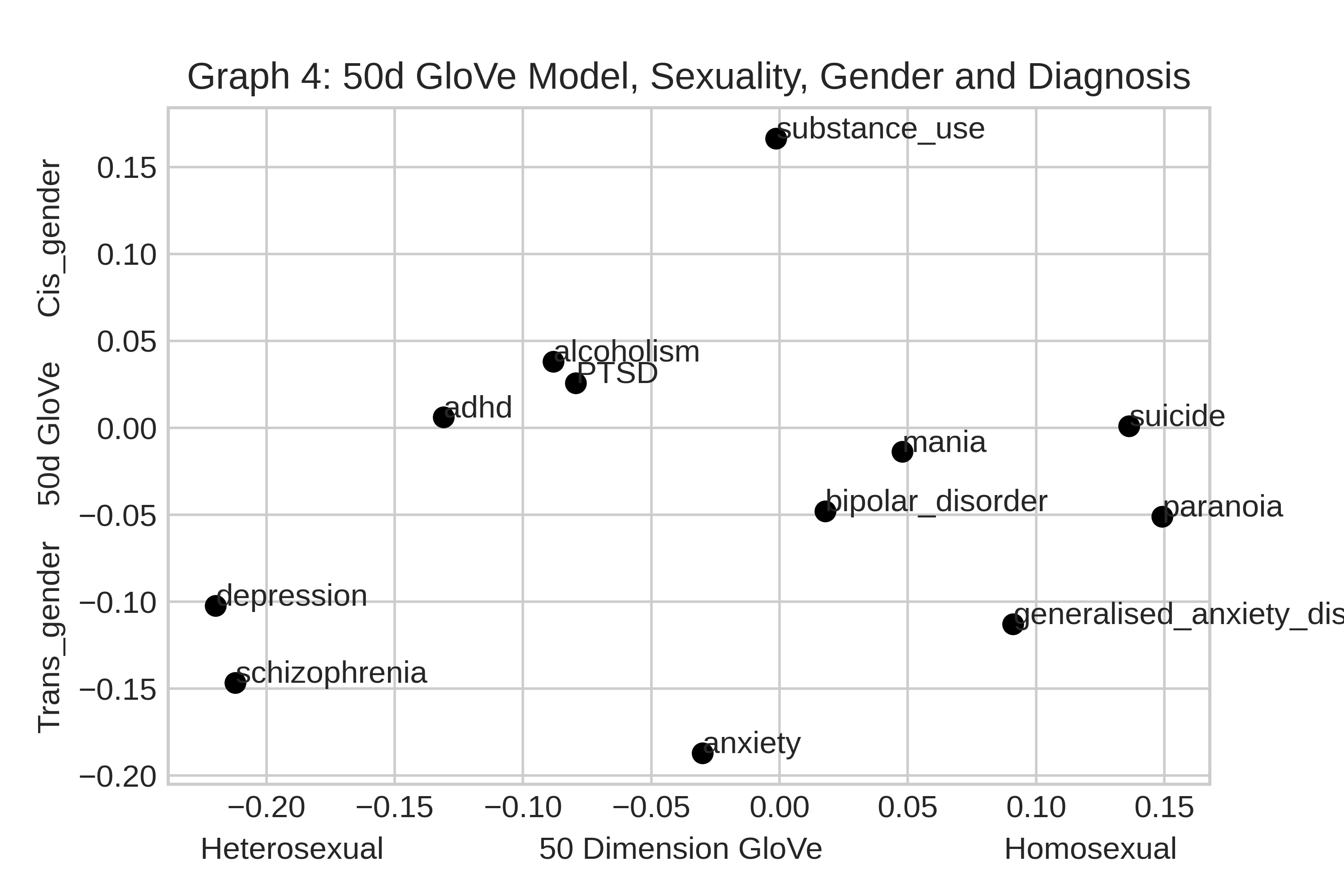
**

**Graph 4.1 (200d): 200 Dimension Sexuality, Gender Bias and Diagnostic Bias (terms ‘heterosexual’, ‘homosexual’, ‘cis_gender’ and ‘trans_gender’).**

Graph 4 illustates the relationships between psychiatric terms and sexuality, and gender labels in the word embeddings. On the X-axis we use the opposing poles "Heterosexual' and 'Homosexual' to look at sexuality, and on the Y-axis we use 'Cis_gender' and 'Trans_gener' to incorporate gender identity.

**
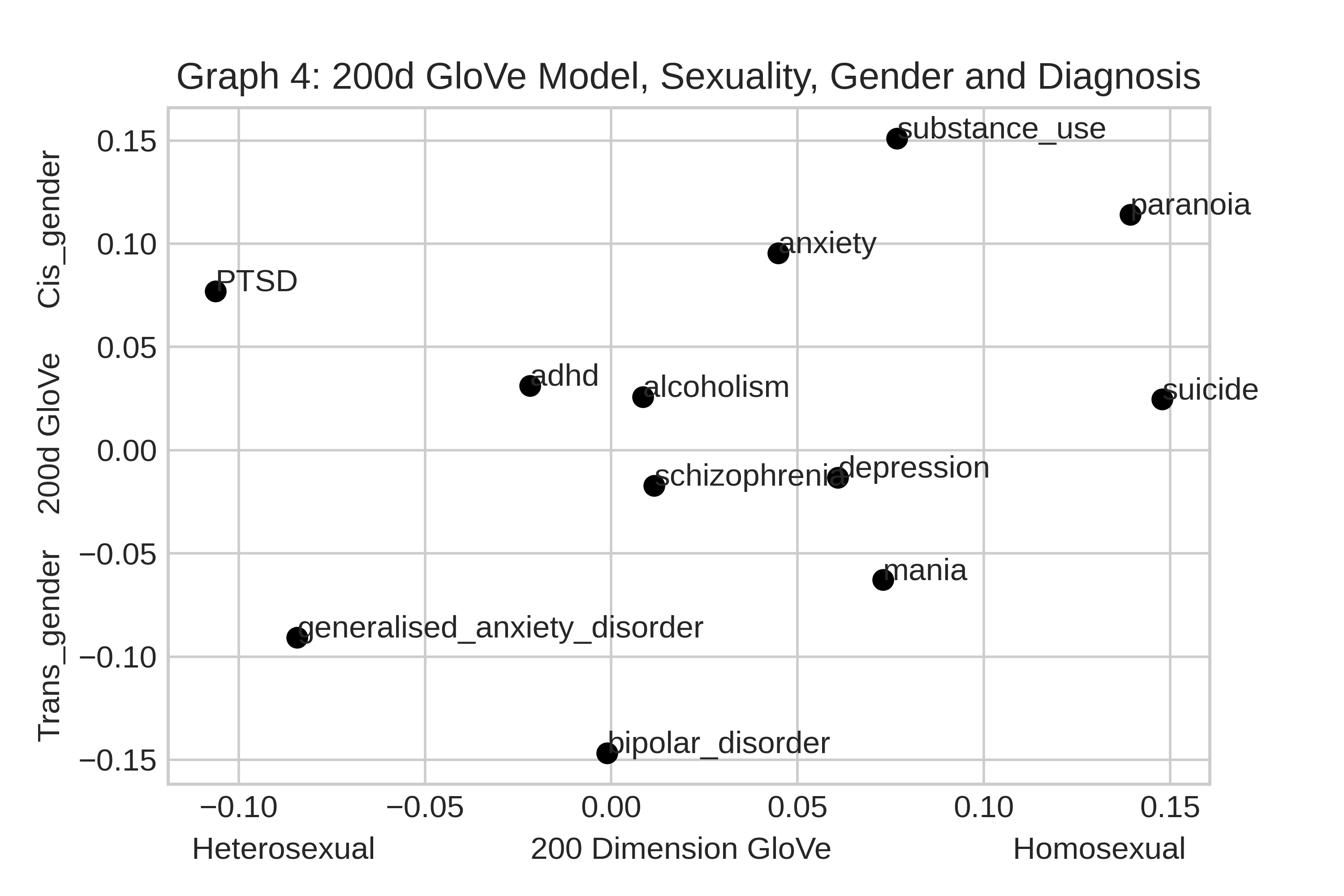
**

**Graph 4.2 (300d): 300 Dimension Sexuality, Gender Bias and Diagnostic Bias (terms ‘heterosexual’, ‘homosexual’, ‘cis_gender’ and ‘trans_gender’).**

**
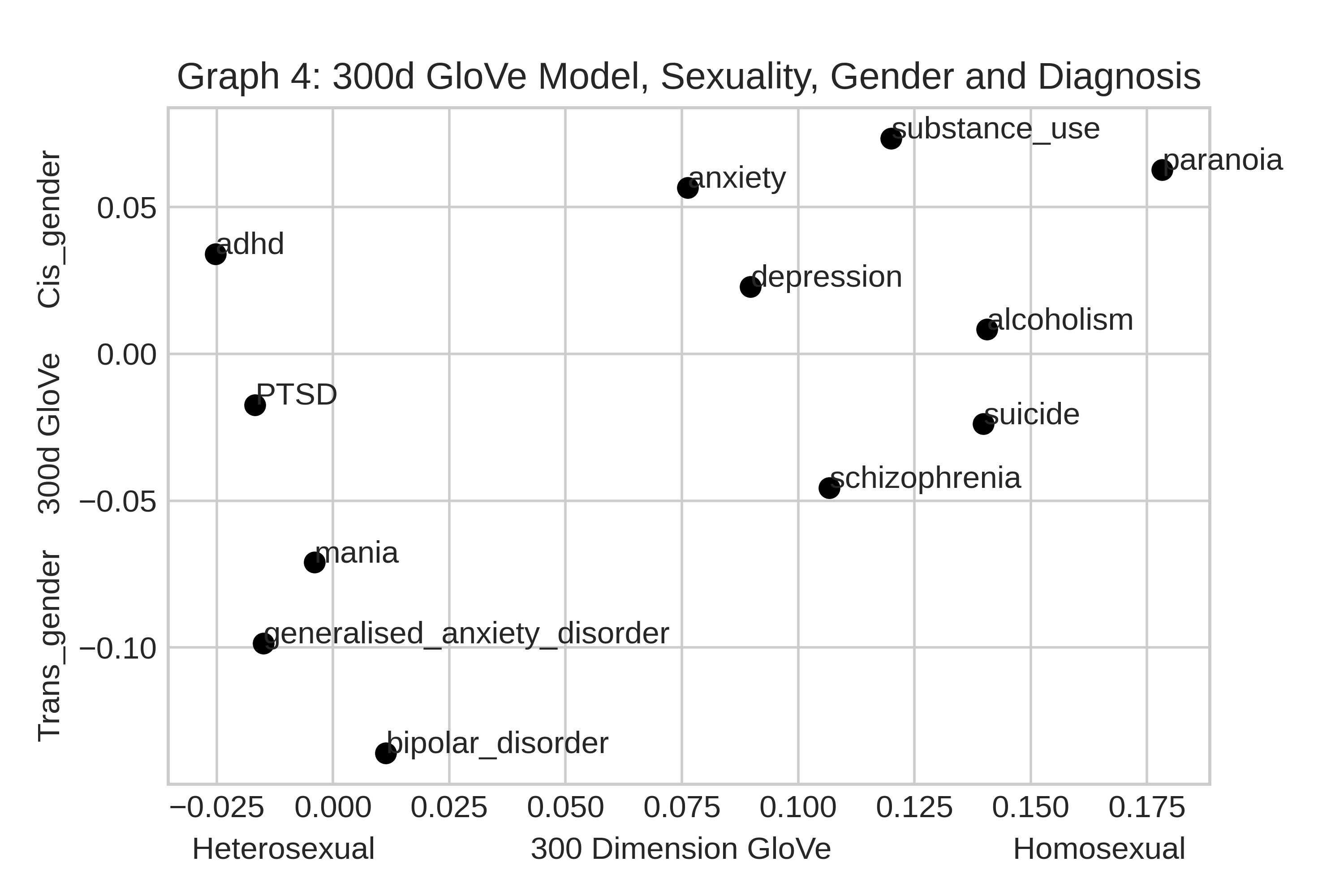
**
